# Permeability-controlled optical modulator with Tri-gate metamaterial: control of permeability on InP-based photonic integration platform

**DOI:** 10.1038/srep08985

**Published:** 2015-03-23

**Authors:** Tomohiro Amemiya, Atsushi Ishikawa, Toru Kanazawa, JoonHyung Kang, Nobuhiko Nishiyama, Yasuyuki Miyamoto, Takuo Tanaka, Shigehisa Arai

**Affiliations:** 1 Quantum Nanoelectronics Research Center, Tokyo Institute of Technology, Tokyo 152-8552, Japan; 2 RIKEN Metamaterials Laboratory, Saitama 351-0198, Japan; 3 Department of Electrical & Electronic Engineering, Okayama University, Okayama 700-8530, Japan; 4 Department of Physical Electronics, Tokyo Institute of Technology, Tokyo 152-8552, Japan; 5 Department of Electrical and Electronic Engineering, Tokyo Institute of Technology, Tokyo 152-8552, Japan; 6 Department of Innovative and Engineered Materials, Tokyo Institute of Technology, Kanagawa 226-8502, Japan; 7 Research Institute for Electronic Science, Hokkaido University, Sapporo 001-0020, Japan

## Abstract

Metamaterials are artificially structured materials that can produce innovative optical functionalities such as negative refractive index, invisibility cloaking, and super-resolution imaging. Combining metamaterials with semiconductors enables us to develop novel optoelectronic devices based on the new concept of operation. Here we report the first experimental demonstration of a *permeability-controlled* waveguide optical modulator consisting of an InGaAsP/InP Mach-Zehnder interferometer with ‘tri-gate’ metamaterial attached on its arms. The tri-gate metamaterial consists of metal resonator arrays and triple-gate field effect elements. It changes its permeability with a change in the controlling gate voltage, thereby changing the refractive index of the interferometer arm to switch the modulator with an extinction ratio of 6.9 dB at a wavelength of 1.55 μm. The result shows the feasibility of InP-based photonic integrated devices that can produce new functions by controlling their permeability as well as their permittivity.

The most important parameters for photonic devices^1^,^2^,^3^ are the electric permittivity *ε* and the magnetic permeability *μ* of materials used in the device. The relative permeability of every natural material is 1 at optical frequencies because the magnetization of natural materials cannot follow the alternating magnetic field of light. Therefore, we were able to control only one parameter, *permittivity*, in the design of photonic devices. The advent of metamaterials has, however, removed this restriction and enabled us to take steps toward unexplored *ε*-*μ* regions^4^,^5^,^6^,^7^,^8^,^9^,^10^,^11^,^12^. This metamaterial paradigm gives us the opportunity for further development of photonic devices with novel optical functionalities, such as the trapped rainbow light storage^13^,^14^, the invisibility cloaking^15^,^16^,^17^,^18^,^19^,^20^,^21^,^22^,^23^,^24^, and imaging with super resolution^25^,^26^,^27^.

As a novel application of metamaterials, we here show the possibility of controlling the permeability in InP-based photonic platforms. Unlike permittivity, the permeability of III-V semiconductors is difficult to move away from *μ* = 1 at optical frequencies. By using metamaterials, however, we can change the permeability with a controlling signal as described in the following. Changing permeability in addition to permittivity produces large modulation of the refractive index of semiconductors. This facilitates the manipulation of light and the management of photons^28^,^29^, and will bring conventional optical devices into small-sized, high-performance devices for photonic integration.

The important requirements for variable permeability devices for photonic integration are that (i) the device be compatible with a planar waveguide structure on InP-based photonic platforms and that (ii) its permeability change be controlled with an external electric signal. A few methods are considered to create metamaterials with variable magnetic property; leading examples are the split-ring resonator array combined with Micro-Electro Mechanical System (MEMS) actuators^30^ and the Si-based optically controlled modulator with negative-index tuning^31^,^32^. However, neither meets the above-mentioned requirements and can be applied to photonic integrated devices. As a new method, we propose controlling the magnetic property of a metamaterial by using electrically-induced carrier accumulation in InP-based semiconductors. Here we demonstrate an InGaAsP/InP optical modulator that controls light intensity by changing the permeability of its waveguide with a voltage signal. The device has the form of a waveguide, so it can be monolithically integrated with other waveguide-based optical devices.

The permeability-controlled optical modulator we made is shown in Fig. 1(a). It consists of an InGaAsP/InP Mach-Zehnder interferometer (MZI) with ‘tri-gate’ metamaterial (TGM) attached on the MZI arms. The TGM is an array of split-ring resonators (SRRs) combined with triple-gate field effect elements (see Fig. 1(b)). Each SRR is composed of a Ti/Au metal ring formed on the Al_2_O_3_-covered, fin-shaped surface (InGaAs) of the InGaAs arms. The metal ring wraps the fins on three sides to form the triple-gate field effect elements. An individual SRR acts as an LC resonant circuit consisting of an inductor formed by the SRR ring and a capacitor formed by the triple-gated InGaAs fins. If input TE-polarized light has a frequency equal to the LC resonance, a circulating current is induced in the SRRs and produces a magnetic dipole moment in response to the input light. Therefore, the TGM operates as a metamaterial with non-unity permeability.

To control the permeability with an external voltage signal, we placed on the TGM a controlling gate that couples capacitively to the SRRs. In this structure, each SRR acts as a floating triple-gate electrode in addition to its role as an LC resonator. Applying a positive voltage to the controlling gate induces electrons in the fins to change the capacitance and consequently the resonance of the SRRs. Thus, we can control the magnetic response of the SRRs, thereby controlling the permeability of the TGM, with the controlling gate voltage.

We simulated the distribution of induced electron density in the fin using a 3-dimensional TCAD device simulator (see Methods). Figure 2(a) shows the results calculated for controlling gate voltages *V_g_* = 0 V and *V_g_* = 20 V. Figure 2(b) depicts the induced electron density along the vertical center line (red line in the top of Fig. 2(a)) and the horizontal center line (blue line in the top of Fig. 2(a)), with *V_g_* as a parameter. The electron density in the fin is effectively modulated by the controlling gate voltage because of the triple-gate structure. Thus, we can control the resonance of the SRRs, thereby changing the permeability of the TGM. The intrinsic speed of this device is limited by electron-accumulation/extinction speed in the fin in the on-off driving of the gate voltage. Figure 2(c) shows the time dependence of the induced electron density in the centre of the fin calculated with a 3-dimensional TCAD device simulator. Electron accumulation and extinction speeds are 1.5 and 2.0 ps, respectively. This result indicates that the operation speed over 50 GHz can be achieved with this modulator (no traveling-wave electrode will be necessary because a MZ arm length of the device will ultimately become less than 50 μm).

Before fabricating actual modulators, we made a TGM array on an InGaAs/InP wafer and estimated its optical properties by on-wafer measurement at a wavelength of around 1.55 μm (see Fig. 3(a)). The fabrication process was as follows. First, a grid of InGaAs fins was formed on the surface of the wafer using electron-beam lithography and CH_4_/H_2_ reactive ion etching, and then the 10-nm-thick Al_2_O_3_ layer was deposited. Next, square SRR rings (consisting of 10-nm-thick Ti and 30-nm-thick Au) were made using a lift-off process. The triple-gate field effect elements were automatically formed at the crossing points between the SRR rings and fins. After that, a 100-nm-thick SiO_2_ layer was formed on the surface by plasma-enhanced chemical vapour deposition. Figure 3(a) shows tilted scanning electron micrographs of a TGM (before SiO_2_ deposition) with SRR dimensions of (i) outer size of ring = 300 × 300 nm, (2) ring-wire width = 50 nm, (iii) fin width including the Al_2_O_3_ layer = 75 nm, and (iv) fin height = 60 nm. We made several TGMs with different SRR ring sizes.

We measured the transmission spectra of the TGMs for input light travelling normal to the TGM plane, using a Fourier-transform infrared spectrometer (FTIR). To clarify the effect of the interaction between the input light and the SRRs, we measured the ratio of the transmission intensity of the experimental device (with TGMs) to that of the control device (without TGMs) on the same wafer. Figure 3(b) plots the result with the SRR ring size as a parameter. Input light that travels normal to the TGM plane does not magnetically couple to the TGM, leaving only the Mie resonance caused by particle-plasmon resonance on the surface of the SRR metal ring. The Mie resonance frequency increased with decreasing SRR size and reached near-infrared frequencies (≈230 THz).

Figure 3(b) also shows the simulated transmission spectra of each TGM calculated for input light travelling normal to the TGM plane (the same as in the preceding FTIR measurement) and parallel to the TGM plane (the same as in actual device operation) using the finite element method (FEM). In this simulation, the conductivity of the metal ring was defined according to the Drude model, and the conductivity of the InP-based III-V semiconductors with free carriers was calculated by taking into account the band filling (Burstein-Moss effect), band-gap shrinkage, and free-carrier absorption (plasma effect)^33^ (see Methods). For the input light travelling normal to the TGM plane with 300 × 300-nm SRRs, only the Mie resonance of the TGM was observed at about 230 THz (point A), which is consistent with our FTIR measurements. In contrast, the input light travelling parallel to the TGM plane shows an LC resonance at 185 THz (point C) in addition to the Mie resonance at 230 THz (point B). This means that, in actual devices, the TGM will interact magnetically with input TE-polarized light to produce non-unity permeability at optical communication wavelengths (around 193 THz). The theoretical and experimental data are consistent for normal incident light, and therefore we can be convinced that the calculated results for parallel incident light will also consistent with the actual characteristics of the metamaterial.

Figure 3(c) shows the distribution of the electric and magnetic fields around the SRR at each resonant frequency (points A, B and C), and the distribution of vector fields is also visualized by a red arrow. For input light travelling normal to the TGM plane (Simulation 1), an electric field is present parallel to the two symmetric sides of the split ring, and the current in the perpendicular directions was negligible (A and B). In contrast, for TE-mode light travelling parallel to the TGM plane (Simulation 2), a magnetic field perpendicular to the axis of the split-ring created a circulating current via the charge accumulation at the gap. Induction magnetic field is also generated inside the TGM ring to cancel the external magnetic field.

On the basis of these results, we designed and fabricated a permeability-controlled optical modulator as follows. Three layers, i.e.; an InGaAsP core layer (λ*_g_* = 1.22 μm, 200 nm thick), an n-type InP cladding layer (350 nm thick, 5 × 10^17^/cm^3^), and an undoped InGaAs fin channel layer (50 nm thick), were grown in this order on a (100)-oriented n-type InP substrate by means of metal-organic vapour phase epitaxy. Then, TGMs were formed on this surface using the procedure described previously. After that, MZI patterns were formed using electron-beam lithography and CH_4_/H_2_ reactive ion etching. Finally, the device was covered entirely with a SiO_2_ layer, and electrodes were deposited on the top of the device and on the InP cladding layer (see Fig. 1(b)). The top electrode is the controlling gate. The gate voltage is applied between the two electrodes.

Figure 4(a) shows the plan view of the fabricated optical modulator observed with an optical microscope. A TGM is formed on one arm of each MZI. We hereafter call this arm an ‘active TGM arm’. The length of the active TGM arm (i.e.; that of the TGM along the arm) was set to 200 μm. To maintain the balance between the two arms, we formed a dummy TGM on the other arm of each MZI. The dummy TGM has almost the same structure as that of the active TGM arm but has no magnetic interaction with 1.55 μm light. Figure 4(b) shows oblique views of the active and dummy TGMs before the MZI pattern was formed. The dummy TGM differs from the active TGM in the number of SRR ring cuts (4 cuts for the active, whereas 2 cuts for the dummy). Figure 4(c) shows the simulated transmission spectra of the active and dummy TGMs calculated for incident light parallel to the TGM array using the FEM. Unlike the active TGM, the dummy shows no magnetic resonance (*LC* resonance) because its resonance frequency is far lower than optical communication frequencies (<100 THz).

In this MZI device, the phase difference Δ*φ* between two arms for a given controlling gate voltage is given by
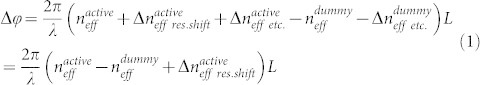
where *λ* is the wavelength of the input light, *L* is the length of the TGM arm, 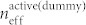 is the effective refractive index of the active (or dummy) TGM arm at gate voltage = 0, 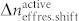 is the change in the refractive index of the active TGM arm that is induced under a given gate voltage by the resonance-frequency shift of the TGM, and 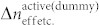 is the change in the refractive index of the active (or dummy) TGM arm that is induced under a given gate voltage by parasitic factors such as accumulated charge carriers. The 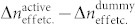 term in Eq. (1) can be ignored because the refractive index change induced by parasitic factors is almost equal for both the active and dummy TGM arms. The phase difference Δ*φ* is therefore determined by 
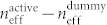
 and 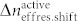. The difference 
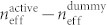
 is produced by the non-unity permeability of the active TGM induced by the interaction between the SRRs and light without a gate voltage, and 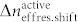 is produced by the change in the permeability of the active TGM produced by a given gate voltage. Thus, the transmission characteristics of the MZI are all related to a change in permeability of the active TGM.

In the actual measurements, TE/TM-polarized light was emitted from a tuneable laser and fed into the device through a polarization controller. The wavelength was changed in the range of 1530–1570 nm. The magnetic field of light was set normal to the TGM plane so that the light would interact magnetically with the TGM array. The output light from the other end of the device was gathered by a lens so that the near-field pattern could be observed. After confirming that the output light was in a single mode, we measured its intensity changes for different controlling gate voltages.

Figure 5(a) shows the transmission characteristics of the device with TGM arm length of 200 μm as a function of the controlling gate voltage, measured for 1550 nm, TE-polarized light. To confirm the TGM-size dependence of the transmission characteristics, we prepared three devices with different SRR-ring sizes of 300 × 300 nm, 350 × 350 nm, and 400 × 400 nm. The transmission intensity changed only in the device with 300 × 300 nm SRRs and increased with controlling gate voltage.

For input light of 1550 nm in wavelength, the 300 × 300-nm TGM interacts magnetically with the light and changes its permeability from 1, whereas the dummy TGM does not interact with the light and maintains its permeability of 1. Consequently, a phase difference occurs between the two arms. If the controlling gate voltage is increased, the TGM deviates from the resonance, and therefore its permeability approaches 1, resulting in a decrease in the phase difference. As a result, the transmission intensity will increase with gate voltage. This was confirmed by our measurements, and an extinction ratio of 6.9 dB was obtained for gate voltage swing from 2.0 to 12.0 V. In this device, the intrinsic propagation loss excluding the measurement system loss was about 20 dB. Most of the loss is caused by a metallic material loss of the TGM array. Strong magnetic response between incident light and the TGM array increases the amount of light absorbed in the TGM thereby increasing the intrinsic loss of the device. It is therefore difficult to reduce the loss of the TGM itself. However, in InP-based photonic devices, it would be possible to reduce total loss by using active waveguides with power gain.

In Fig. 5(a), the modulation in the device saturates at 15–20 V gate voltage. This is because the gap capacitance of the triple-gate SRR changes with carriers induced in the fin by the gate voltage and saturates at a carrier density of about 10^−19^ cm^−3^, corresponding to a gate voltage of 15–20 V (Also see a supplementary material that shows the calculated carrier-density dependence of the refractive index and absorption coefficient of the InGaAs fin at optical frequencies). Consequently, the resonance frequency of the SRRs and therefore the optical properties of the device change with the gate voltage and saturate at 15–20 V gate voltage.

Figure 5(b) shows the transmission characteristics of the device with TGM arm length of 200 μm as a function of the controlling gate voltage, measured for 1550 nm, TM-polarized light. The transmission intensity was almost constant in each device. This is because the TM wave has no magnetic interaction with the ring because its magnetic field is parallel to the ring plane, and therefore no change is produced in permeability. In general, photonic semiconductor integrated devices are used in TE-mode operation because light from laser diodes is TE-polarized. Therefore, the insensitivity to TM waves would not be a problem in practical application.

We also calculated the transmission characteristics of the device. In our simulation, we first calculated complex effective refractive indices of the active and the dummy TGM arms under a given gate voltage (see Methods). Using the calculated effective refractive indices, we performed waveguide analysis based on the fast multipole method (FMM) to evaluate the transmission characteristics of the permeability-controlled MZ modulator. Figure 5(b) shows the simulated transmission intensity of the device with TGMs that have 300 × 300-nm SRRs. The length of the TGM arm was changed as a parameter. The experimental data measured for 100-, 200- and 300-μm arm length are plotted on the simulated curve, and the simulated and experimental data are found to be consistent. The 1000-μm device showed a π or more phase shift in its MZ arm with appropriate gate voltages. The minimum device length needed for a π phase shift was 500 μm. The simulation predicts that a maximum extinction ratio of 15 dB can be expected for the 500-μm device.

We further calculated the complex relative permeability of the TGM arm. Figure 5(c) shows the real part of the relative permeability calculated as a function of light wavelength for gate voltages *V_g_* = 0 V and 20 V. The effective permeability of the active TGM arm exhibits a sharp resonance at about 1620 nm (corresponding to the LC resonance frequency, 185 THz) and, at *V_g_* = 0 V, changes from 0.97 to 1.03 around this frequency (see the red curve). Outside of the resonance region, the effective permeability approaches 1, the same value as that of the dummy TGM arm. If the gate voltage rises from 0 to 20 V, electrons accumulate in the fins, and this produces a blue shift of the resonance frequency of the TGM (see the blue curve). Consequently, the permeability of the active TGM arm at a given optical frequency can be changed with the gate voltage. For example, at 1550 nm (193 THz), the permeability changed from 1.027 to 1.019 with the gate voltage from 0 to 20 V. Thus, the permeability of semiconductor devices can be electrically changed by using the gate-controlled SRR metamaterial. In this way, we can make variable-permeability devices that are compatible with InP-based photonic integration.

In this work, we measured the transmission characteristic of the device only at a wavelength of 1.55 μm because of the restriction caused by the MMI used in the device and the measurement system. However, the simulation in Fig. 5(d) shows that a larger change in permeability can be obtained at differing wavelengths (e.g. 1520 nm and 1590 nm). Using these regions with a modified device structure will enable a higher device performance. As a future target, we calculated the performance of the permeability-controlled modulator when using the maximum permeability change. Figure 6 summarizes the results, i.e., a required MZ arm length for realizing π-phase shift together with a propagation loss of the device as a function of the distance between the InGaAsP core and TGM layer. In our modulator, light traveling along the InGaAsP/InP waveguide extends through the *n*-InP cladding layer into the TGM layer and interacts to ensure magnetic response (see Fig. 6(a)). Therefore, the thickness of the *n*-InP cladding layer greatly affects performance of the modulator.

As shown in Fig. 6(b), extremely short device length (<50 μm) can be realized because changing permeability in addition to permittivity produces large modulation of the refractive index of semiconductors. If we set the interaction distance to be 300 nm, the π-phase shift can be obtained with the MZ arm length of 35 μm with the propagation loss lower than 0.15 dB/μm. This will transform conventional optical modulators (e.g. electroabsorption modulator) into small-sized, high-performance devices for photonic integration.

## Methods

### Supporting information for numerical calculation

We used the Silvaco Atlas simulator to calculate a three-dimensional carrier distribution in the TGM structure (Ti/Au SRR rings formed on the InGaAs/InP fins) under a given gate bias. For the three-dimensional analysis, we took into consideration the Poisson equation, electron and hole continuity equation, parallel electric field-dependent mobility model, concentration-dependent carrier mobility model, Shockley-Read-Hall (SRH) recombination model, material-dependent band parameter model, and Fermi-Dirac statistics model.

We then calculated the carrier-induced changes in the refractive index *n* and absorption loss *α* in each region of the multiple fins. In this simulation, three effects, i.e.; the band filling, bandgap shrinkage, and free-carrier absorption, were assumed to make substantial contributions to the total changes in the refractive index and absorption loss. In the case of band filling, there is a finite probability that a state in the conduction band is occupied by an electron and/or a state in the valence band is empty. Thus, the band-filling-induced change in absorption is given by 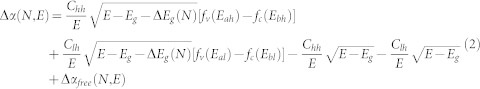
where *E* = *hω*/2*π* is the photon energy; *N* is the concentration of free electrons; *E_g_* is the bandgap energy; *C_hh_* and *C_lh_* are constants involving materials parameters, matrix elements between periodic parts of the Bloch states at the band edges, and fundamental constants for heavy and light holes^34^; *f_c_*(*E_b_*) is the probability of a conduction band state of energy *E_b_* being occupied by an electron; and *f_v_*(*E_a_*) is the probability of a valence band state of energy *E_a_* being occupied by an electron.

For a given photon energy, the values of *E_a_* and *E_b_* are uniquely defined. Here we also introduce the bandgap shrinkage and free-carrier absorption, which cause a rigid translation of the absorption curve. The bandgap shrinkage Δ*E_g_*(*N*) is given by^35^
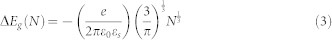
where *ε_0_* is the permittivity of free space and *ε_s_* is the relative static dielectric constant of the semiconductor. On the other hand, an expression for the free-carrier absorption Δ*α*_free_(*N,E*) is
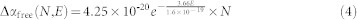


The real and imaginary parts of the refractive index are related by the Kramers-Kronig integrals. Therefore, the change in refractive index Δ*n* was finally calculated by applying the following integral to Δ*α*:
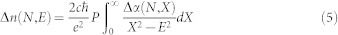
where *P* indicates the principal value of the integral.

Using the obtained refractive index and absorption loss in each region of the TGM under a gate bias, we calculated the transmission characteristics with the aid of the commercial finite-element package COMSOL Multiphysics. In this simulation, the conductivity of the metal was defined according to the Drude model, and the eigenvalue solver was used to find modes of the device.

**Figure 3:**
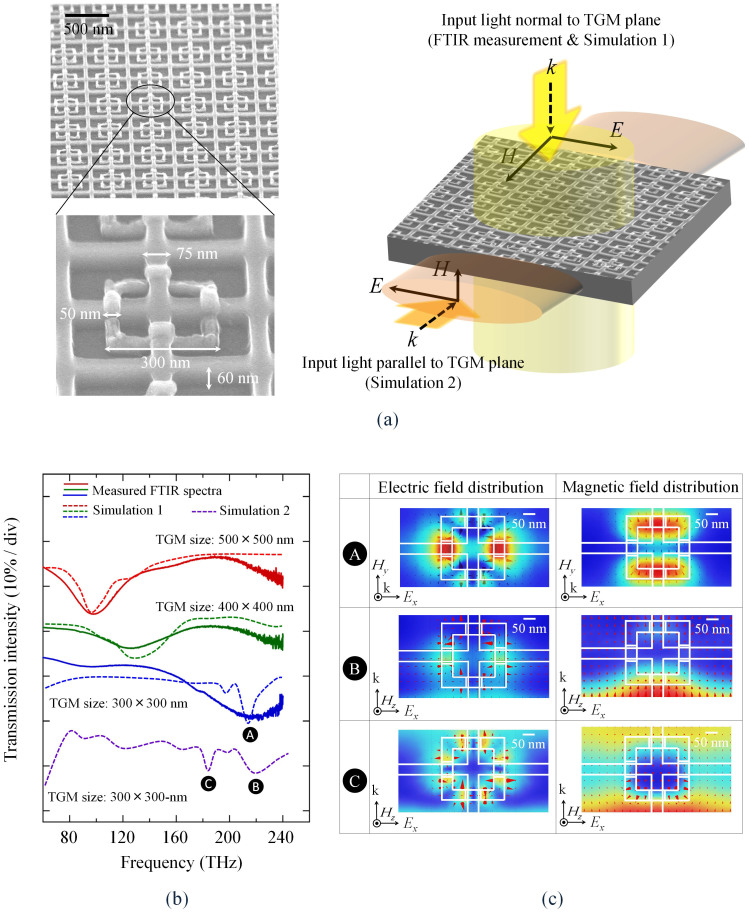
Optical measurement of TGM. (a) TGM observed by scanning electron microscopy: oblique view of a TGM formed on a InGaAs/InP wafer and enlarged view of an individual TGM unit (left). Three-dimensional view of a TGM on InGaAs/InP wafer, with two light waves travelling normal and parallel to the TGM plane (right). (b) Transmission spectra for normal light with the SRR ring size as a parameter, with simulation results of a 300 × 300, 400 × 400, 500 × 500 nm SRR rings calculated for normal light (Simulation 1) and a 300 × 300 nm SRR ring calculated for parallel light (Simulation 2). (c) Electric and magnetic field distributions around the SRR at each resonant frequency (A, B and C). The distribution of vector field is also visualized by a red arrow.

**Figure 5:**
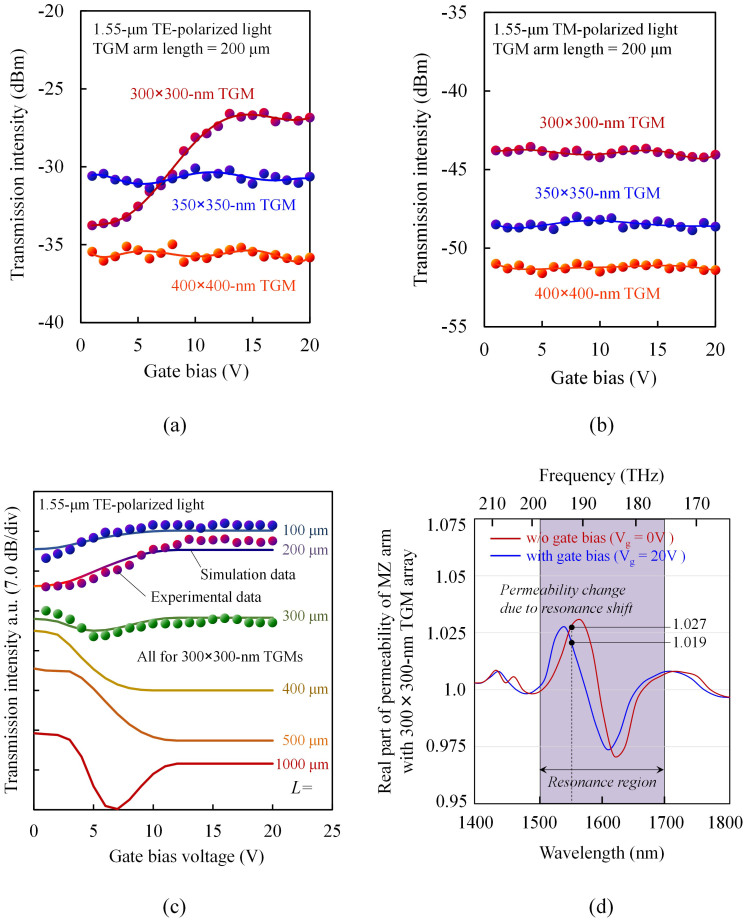
Operation of permeability-controlled optical modulator. (a) Transmission intensity as a function of gate voltage measured for 1550 nm TE-polarized parallel light. (b) Transmission intensity as a function of gate voltage measured for 1550 nm TM-polarized parallel light. Results for three TGMs with different SRR ring sizes of 300 × 300 nm, 350 × 350 nm, and 400 × 400 nm are plotted. (c) Transmission intensity change as a function of gate voltage calculated for different lengths of the TGM array on the MZI arm. Measured result for length of 100, 200, and 300 μm is also plotted. (d) Real parts of the relative permeability of the 300 × 300-nm TGM arm calculated as a function of wavelength, with gate voltages of 0 V and 20 V.

To obtain transmission spectra shown in Fig. 3(b), we calculated the electromagnetic wave propagation over a cubic unit cell consisting of an individual TGM on GaInAs/InP. The extremities of the calculation region were defined as a perfect electric conductor (PEC) and a perfect magnetic conductor (PMC) to imitate the mutual interactions of each TGM. To obtain the transmission characteristics for the actual modulator, as shown in Fig. 5(c), we first examined the complex effective refractive index from the calculated *S*-parameters for one period of the InGaAsP/InP waveguide with an active/dummy TGM. In the simulation for determining the *S*-parameters, the extremities of the calculation region were given scattering properties to imitate the necessary open boundary conditions. After that, using the complex effective refractive indices, we conducted a waveguide analysis based on the FMM to evaluate the transmission characteristics of the permeability-controlled MZ modulator.

## Author Contributions

T.A., A.I., T.T. and S.A. conceived and designed the experiments. T.A. fabricated the samples. T.A., T.K. and A.I. carried out the measurements. T.K. and Y.M. performed the simulations. N.N. contributed to the development of the measurement set-ups. J.K. contributed to the sample fabrication. T.A., T.K. and A.I. analysed the data. T.A., A.I., T.T. and S.A. wrote the paper. All authors discussed the results and commented on the manuscript.

## Supplementary Material

Supplementary Information Supplementary material

## Figures and Tables

**Figure 1:**
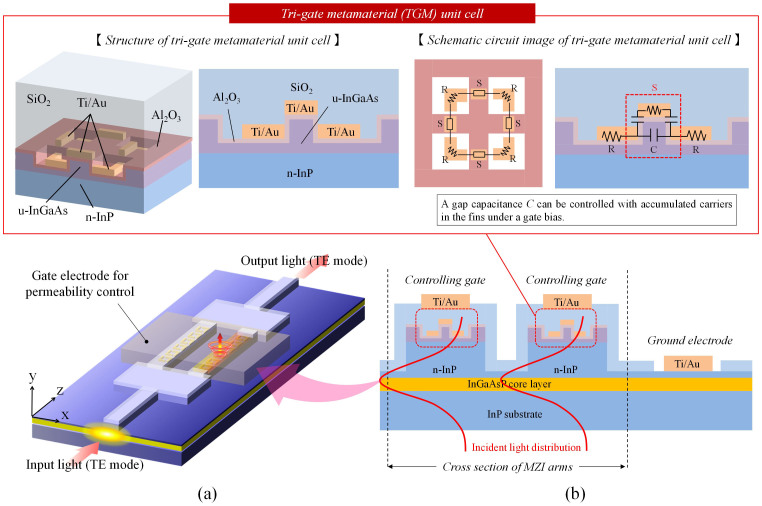
Permeability-controlled optical modulator using metamaterial. (a) Structure of the device consisting of an InGaAsP/InP Mach-Zehnder interferometer with tri-gate metamaterials. (b) Unit cell of tri-gate metamaterials consisting of a SRR and triple-gate field effect elements (top), and cross section of a Mach-Zehnder arm with tri-gate metamaterial (bottom).

**Figure 2:**
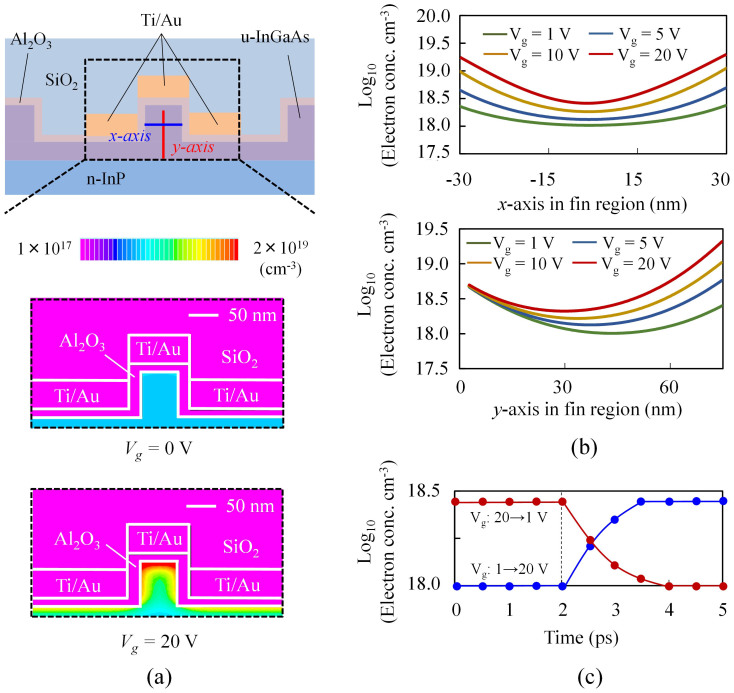
Electron density modulation in triple-gate structure simulated with three-dimensional TCAD. (a) Electron density induced in the InGaAs fin at controlling gate voltages *V_g_* = 0 V and 20 V. (b) Accumulated electron densities along the horizontal center line (x direction, blue line in Fig. 2(a)) and vertical center line (y direction, red line in Fig. 2(a)) of the fin, with the controlling gate voltage as a parameter. (c) Time dependence of carrier accumulation (blue line)/extinction (red line) in the centre of the triple-gate field effect element in the on-off driving of the gate voltage (switched at t = 2 ps).

**Figure 4:**
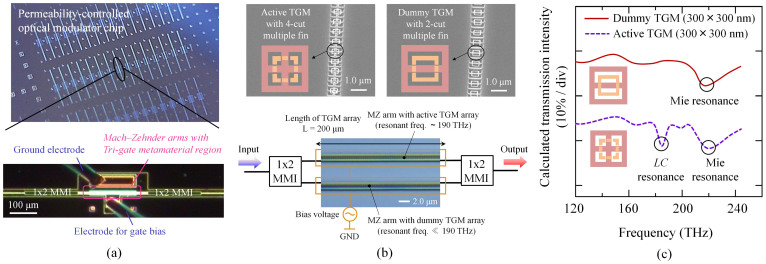
Permeability-controlled optical modulator. (a) Plan view observed with an optical microscope. (b) Oblique view of active and dummy TGM arrays (before MZI pattern formation) observed by scanning electron microscopy. The length of the TGM array along the arm was set to 200 μm. The phase difference between the two arms is dependent only on the permeability change of the active TGM array and is independent of parasitic factors. (c) Simulated transmission spectra of the active and dummy TGMs calculated for incident light parallel to the TGM array using the FEM.

**Figure 6:**
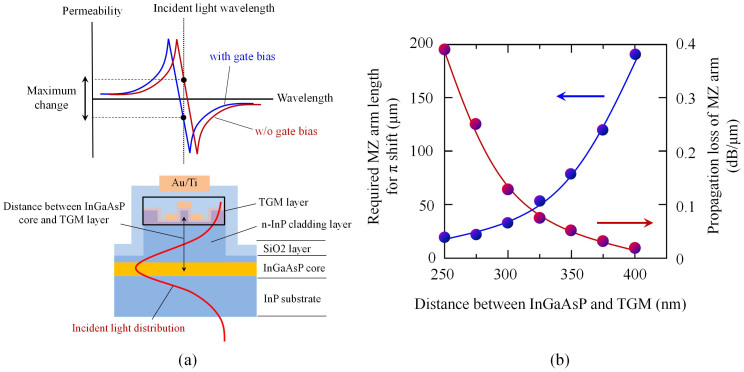
Towards higher performance of permeability-controlled modulator. (a) Schematic image of permeability change and cross sectional image of device. (b) Required MZ arm length for realizing π-phase shift together with a propagation loss of the device as a function of the distance between the InGaAsP core and TGM layer.
