# Supplementary material for: Permeability-controlled optical modulator with Tri-gate metamaterial: control of permeability on InP-based photonic integration platform
Source: Sci Rep. 2015 Mar 23;5:8985. doi: 10.1038/srep08985 (PMC4369730; doi:10.1038/srep08985)
Supplement: Supplementary Information — Supplementary material [file srep08985-s1.doc]

**SUPPLEMENTARY INFORMATION**

**Permeability-controlled optical modulator with Tri-gate metamaterial: control of permeability on InP-based photonic integration platform**

**Tomohiro Amemiya^1,2*^, Atsushi Ishikawa^2,3^, Toru Kanazawa^4^, JoonHyung Kang^5^, Nobuhiko Nishiyama^5^, Yasuyuki Miyamoto^4^, Takuo Tanaka^2,6,7^ & Shigehisa Arai^1,5^**

*^1^Quantum Nanoelectronics Research Center, Tokyo Institute of Technology, Tokyo 152-8552, Japan,*

*^2^RIKEN Metamaterials Laboratory, Saitama 351-0198, Japan,*

*^3^Department of Electrical & Electronic Engineering, Okayama University, Okayama 700-8530, Japan,*

*^4^Department of Physical Electronics, Tokyo Institute of Technology, Tokyo 152-8552, Japan,*

*^5^Department of Electrical and Electronic Engineering, Tokyo Institute of Technology, Tokyo 152-8552, Japan,*

*^6^Department of Innovative and Engineered Materials, Tokyo Institute of Technology, Kanagawa 226-8502, Japan,*

*^7^Research Institute for Electronic Science, Hokkaido University, Sapporo 001-0020, Japan.*

**e-mail:* [*amemiya.t.ab@m.titech.ac.jp*](mailto:amemiya.t.ab@m.titech.ac.jp)

SI 1: Carrier-induced changes in the refractive index and absorption loss of the InGaAs fin


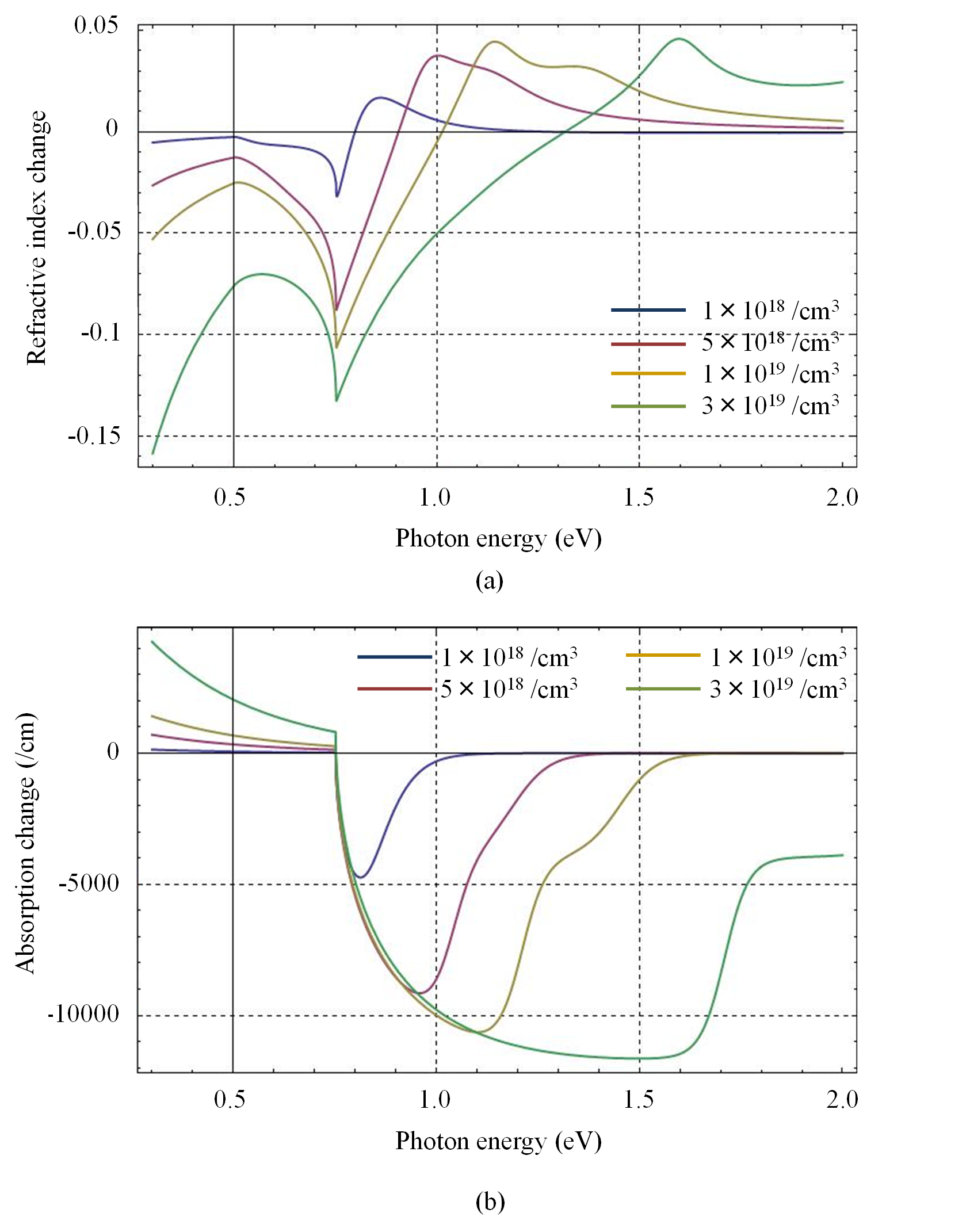


FIG. 1. Calculated the carrier-induced changes in the refractive index (a) and absorption loss (b) of the InGaAs fin at optical frequencies. In this simulation, three effects, i.e.; the band filling, bandgap shrinkage, and free-carrier absorption, were assumed to make substantial contributions to the total changes in the refractive index and absorption loss.
